# Plasma copeptin levels are inversely associated with intima-media-thickness in men: the population-based KORA F4 study

**DOI:** 10.1186/1475-2840-12-168

**Published:** 2013-11-11

**Authors:** Cornelia Then, Bernd Kowall, Andreas Lechner, Christa Meisinger, Margit Heier, Wolfgang Koenig, Annette Peters, Joachim Thiery, Wolfgang Rathmann, Jochen Seissler

**Affiliations:** 1 Medizinische Klinik und Poliklinik IV, Diabetes Zentrum - Campus Innenstadt, Klinikum der Ludwig-Maximilians-Universität, Ziemssenstrasse 1, 80336, Munich, Germany; 2 Clinical Cooperation Group Diabetes, Ludwig-Maximilians-Universität München and Helmholtz Zentrum München, Munich, Germany; 3 German Diabetes Center, Leibniz Institute at Heinrich Heine University Düsseldorf, Institute of Biometrics and Epidemiology, Düsseldorf, Germany; 4 Institute of Epidemiology II, Helmholtz Zentrum München – German Research Center for Environmental Health (GmbH), Neuherberg, Germany; 5 Research Unit of Molecular Epidemiology, German Research Center for Environmental Health, Neuherberg, Germany; 6 Department of Internal Medicine II – Cardiology, University of Ulm, Medical Centre, Ulm, Germany; 7 Institute of Laboratory Medicine, Clinical Chemistry and Molecular Diagnostics, University Hospital Leipzig, Leipzig, Germany

**Keywords:** Intima-media thickness, IMT, Atherosclerosis, Copeptin, AVP, Vasopressin, Diabetes

## Abstract

**Background:**

Elevated plasma preprovasopressin (copeptin) levels are associated with cardiovascular complications as well as with an increased risk for type 2 diabetes (T2D). Here, we studied, whether plasma copeptin is related to carotid intima-media thickness (IMT), a measure of early atherosclerosis, and may thus be one explanation for the high cardiovascular risk in T2D.

**Methods:**

Plasma concentrations of copeptin and IMT of the common carotid artery were determined in 1275 participants of the population-based KORA F4 study. We used linear regression models to investigate associations between copeptin levels and IMT.

**Results:**

In the whole study group, copeptin levels were not significantly associated with IMT after adjustment for age and sex. Copeptin and IMT were significantly inversely associated after multivariable adjustment in the total cohort (β = -0.020 mm, 95% CI: -0.037 mm; -0.003 mm), in men (β = -0.035 mm, 95% CI: -0.061 mm; -0.009 mm) and in study participants with prediabetes (β = -0.041 mm, 95% CI: -0.078 mm; -0.005 mm) comparing quartile 4 vs quartile 1. The negative association of copeptin and IMT in men was present after adjustment for age alone. In women and patients with T2D, copeptin was not significantly associated with IMT.

**Conclusions:**

Plasma copeptin was not associated with an increased IMT in our study cohort. In contrast, copeptin levels were related to a lower IMT in men and subjects with prediabetes, suggesting that elevated copeptin concentrations do not exert proatherogenic effects on carotid arteries.

## Background

The neurohormone arginine vasopressin (AVP) is secreted from the posterior pituitary in response to osmolality, hemodynamic changes and acute and chronic stress [1,2]. AVP targets various vasopressin receptors (VR), promoting antidiuretic and antinatriuretic effects via V2R and mediating vasoconstriction by binding to V1aR on vascular smooth muscle cells [2]. Besides these well-known functions, AVP has multiple metabolic effects: AVP activates release of adrenocorticotropic hormone from the anterior hypophysis (V2R), mediates hepatic gluconeogenesis and glycogenolysis through V1aR and stimulates glucagon and insulin secretion from pancreatic islets (V3R). In addition, AVP exerts a complex influence on the regulation of lipid metabolisms [2].

Plasma AVP is unstable and rapidly cleared from the plasma, preventing reliable measurements. Copeptin, the stable C-terminal part of the AVP precursor molecule, is cleaved from pro-AVP during processing and is secreted in an equimolar concentration with AVP. The development of assays for copeptin determination has made it possible to use copeptin as a reliable surrogate for AVP measurements [3].

Elevated copeptin levels are associated with the metabolic syndrome [4,5], with an increased risk for type 2 diabetes (T2D) and with overt T2D [6,7]. Furthermore, plasma copeptin levels are increased in heart failure [8] and the acute coronary syndrome [9,10] in both non-diabetic subjects and in patients with T2D [11-13]. Increased copeptin concentrations are related to a higher mortality in critically ill patients and provide prognostic implications in patients with end stage renal disease [14], heart failure [15], myocardial infarction [16] and stroke [17,18]. In addition, copeptin has been shown to be associated with all-cause mortality (hazard ratio 1.22) and cardiovascular events (hazard ratio 1.17) in diabetic patients [19]. Thus, it may be possible that increased plasma copeptin concentrations are involved in the development of premature atherosclerosis.

Measurement of intima-media thickness (IMT) of the common carotid artery by high resolution transcutaneous ultrasound is an established non-invasive measure for the early diagnosis of atherosclerosis and carotid plaque has been consistently correlated with atherosclerotic vascular lesions and with an increased risk for stroke and myocardial infarction independently of traditional vascular risk factors [20-23]. Therefore, we sought to evaluate the association of copeptin with IMT in a community-based population. The association of plasma copeptin with both cardiovascular disease [9,10] and T2D [6,7] prompted us to stratify the study cohort according to categories of glucose tolerance in order to examine a possible contribution of elevated copeptin to the high cardiovascular risk in diabetes. Because previous studies have shown significant differences in plasma copeptin values in women and men [4,5,7,24], we also included gender-specific analyses.

## Methods

### Study population

The KORA (Cooperative Health Research in the Region of Augsburg, southern Germany) F4 study is a population-based cohort of 3080 subjects (1486 men, 1594 women) aged 32–81 years surveyed between 2006 and 2008 (follow-up study of the KORA S4 survey conducted in 1999–2001). Standardized sampling methods and data collection (medical history, medication, anthropometric measurements, blood pressure) have been described in detail elsewhere [25,26]. All study participants gave written informed consent and the study was approved by the Ethics Committee of the Bavarian Medical Association. IMT was measured in all study participants who agreed to undergo ultrasound examination (n = 2663). By study design, samples from 50% of the study participants were randomly selected for plasma copeptin measurement (n = 1596). All variables required for the current analyses were available in 1275 study participants. Patients were assigned to one of three groups (normal glucose tolerance (NGT), prediabetes or T2D) according to categories of glucose tolerance. Criteria for known diabetes were a validated physician’s diagnosis or current use of glucose-lowering agents. After an overnight fasting period, all non-diabetic participants underwent a standard 75 g oral glucose tolerance test. Newly diagnosed diabetes, impaired glucose tolerance, impaired fasting glucose and NGT were defined according to the 1999 World Health Organization diagnostic criteria based on both fasting and post-challenge glucose values (≥ 7.0 mmol/l fasting or ≥ 11.1 mmol/l 2 h glucose) [27]. Prediabetes was defined as increased fasting plasma glucose (> 6.1 and < 7.0 mmol/l) and/or impaired glucose tolerance (2 h glucose 7.8 - <11.1 mmol/l). Hypertension was defined as systolic blood pressure ≥ 140 mmHg, diastolic blood pressure ≥ 90 mmHg or known hypertension with use of anti-hypertensive drugs.

### Ultrasound and measurement of IMT

Ultrasound measurements of common carotid arteries (CCA) were performed by two certified investigators as described recently [28]. Briefly, optimal images of the right and left CCA far wall were recorded on DVD videotapes. IMT measurements were performed off-line over a length of 10 mm beginning at 0–5 mm of the dilatation of the distal CCA using an automated edge detection reading system (Prowin software, Medical Technologies International, USA). We used the average of the measurements of the left and right CCA to calculate mean artery thickness of the distal CCA. One certified reader measured all IMT scans.

### Laboratory measurements

Blood was collected without stasis and was kept at 4°C until centrifugation. All blood parameters, except for 2-h glucose, were based on fasting blood samples. Plasma samples were stored at -80°C until assayed and there was only one freeze-thaw cycle before determination of MR-proANP. Measurements of blood glucose, HbA1c, total cholesterol, high-density lipoprotein (HDL) and low-density lipoprotein (LDL) cholesterol, triglycerides, serum creatinine and high sensitive C-reactive protein (hsCRP) were performed as described previously [29]. Glomerular filtration rate was calculated using the MDRD equation (eGFR).

Plasma concentrations of copeptin were measured by a validated sandwich fluoroimmunoassay (BRAHMS, Hennigsdorf/Berlin, Germany) using the automated system B.R.A.H.M.S KRYPTOR as described in detail elsewhere [3]. The lower detection limits was 0.4 pmol/l. Intra- and inter-assay coefficients of variation were 5.9% and 8.9%, respectively.

### Statistical analyses

Baseline characteristics were compared between subjects with NGT, prediabetes, and T2D using F-tests in case of normally distributed variables. For log-normal variables, F-tests were performed on a log-scale. Logistic regression models were used to compare binomial proportions. All comparisons were adjusted for age and sex. Spearman correlation coefficients were calculated between IMT and copeptin, respectively, and conventional cardiovascular risk factors.

In linear regression models, the association between quartiles of copeptin and IMT was assessed in different models: (1) without adjustment, (2) with adjustment for age and sex, (3) for age, sex, and body mass index (BMI), and (4) age, sex, BMI, waist circumference (continuous), hypertension (≥ 140/90 mm Hg or antihypertensive medication), HDL cholesterol (continuous), LDL cholesterol (continuous), triglycerides (continuous), prediabetes (yes/no), T2D (yes/no) former myocardial infarction or stroke, smoking behaviour (active/former/never), alcohol consumption (never/moderate/high), physical activity (high/low), hsCRP and eGFR. These models were fitted for the whole study group, for women and men separately, and for subjects with and without impaired glucose metabolism. Furthermore, the association between copeptin and IMT was determined using copeptin as continuous variable.

As the assumptions of normally distributed residuals and of homoscedasticity were not fulfilled when IMT was the dependent variable in linear regression models, we additionally used ln(IMT) as dependent variable in linear regression models. The level of statistical significance was set at 5%. All analyses were performed using the SAS version 9.2 (SAS institute, Cary, NC, USA).

## Results

### Characteristics of the study population

Characteristics of 1275 study participants are given in Table 1. Stratified analysis in men and women revealed higher median crude copeptin levels in men (women: 7.5 (25^th^ percentile 7.4; 75^th^ percentile 13.7) pmol/l; men: 10.1 (25^th^ percentile 7.4; 75^th^ percentile 13.7) pmol/l; p < 0.0001). The copeptin levels in the respective quartiles are shown in Additional file 1: Table S1. There were also sex-specific differences in crude IMT (increased IMT by 0.043 ± 0.008 mm in men, p < 0.0001). Both, copeptin and IMT were related with conventional cardiovascular risk factors. Age, BMI, waist circumference, systolic and diastolic blood pressure, triglycerides, HDL cholesterol, hsCRP and eGFR were all related to plasma copeptin levels in the total study cohort (Table 2). Age (r = 0.22 in men and 0.16 in women) and declining kidney function (eGFR; r = -0.20 in men and -0.16 in women) were significantly associated with copeptin levels in both genders, whereas BMI (r = 0.14), waist circumference (r = 0.13), systolic blood pressure (r = 0.08), triglycerides (r = 0.08) and hsCRP (r = 0.18) were significantly related to copeptin only in women (Table 2). In the total study cohort, IMT values were significantly correlated to age, BMI, LDL cholesterol, HDL cholesterol, triglycerides, systolic blood pressure, waist circumference, disturbed glucose tolerance, eGFR, and hsCRP (p < 0.0001 for all, data not shown). Diastolic blood pressure was only slightly associated with IMT (r = 0.05, p = 0.05).

**Table 1:** **Characteristics of 1275 study participants according to categories of glucose regulation: the KORA F4 study**^**a**^

	**All subjects**	**NGT**	**Prediabetes**	**T2D**	**p**^**b**^
**n**	**1275**	**911**	**224**	**140**	**-**
Age (years)	56.6 ± 12.7	53.4 ± 12.2	63.6 ± 10.5	66.1 ± 8.6	0.001^#^
Sex (male) (n (%))	624 (48.9)	419 (46.0)	120 (53.6)	85 (60.7)	0.003^###^
BMI (kg/m^2^)	27.4 ± 4.5	26.5 ± 4.2	29.4 ± 4.3	30.6 ± 4.4	0.001^#^
Waist (cm)	93.3 ± 13.6	90.2 ± 12.9	99.4 ± 11.9	103.9 ±11.7	0.001^#^
Systolic blood pressure (mm Hg)	122.1 ± 17.7	119.0 ± 16.7	128.9 ± 18.1	131.3 ± 16.7	0.001^#^
Diastolic blood pressure (mm Hg)	75.1 ± 9.6	74.8 ± 9.4	76.8 ± 9.9	74.7 ± 10.2	0.003^#^
HDL cholesterol (mmol/l)	1.47 ± 0.37	1.51 ± 0.38	1.41 ± 0.35	1.29 ± 0.29	0.001^#^
LDL cholesterol (mmol/l)	3.52 ± 0.88	3.49 ± 0.86	3.80 ± 0.92	3.28 ± 0.85	0.001^#^
Triglycerides (mmol/l)	102.0 (71.0,149.0)	93.0 (65.0; 136.0)	126.0 (93.0; 172.5)	130.5 (92.0; 206.0)	0.001^##^
hsCRP (mg/l)	1.10 (0.54; 2.42)	0.935 (0.46; 1.95)	1.71 (0.92; 3.40)	1.70 (0.86; 3.76)	0.001^##^
Estimated GFR (ml/min)	84.0 ± 18.2	85.8 ± 17.6	81.3 ± 18.2	76.8 ± 19.8	0.12^#^
Former stroke or MI (n (%))	58 (4.6)	30 (3.3)	10 (4.5)	18 (12.9)	0.03^###^
Active or former smoker (n (%))	728 (57.1)	534 (58.6)	110 (49.1)	84 (60.0)	0.049^###^
High alcohol consumption (n (%))^c^	249 (19.5)	179 (19.7)	42 (18.8)	28 (20.0)	0.92^###^
Hypertension (n (%))^d^	500 (39.3)	265 (29.1)	127 (56.7)	108 (77.1)	0.001^###^
Physically inactive (n (%))^e^	529 (41.5)	341 (37.4)	110 (49.1)	78 (55.7)	0.001^###^
Copeptin (pmol/l)					
Mean ± SD	10.46 ± 16.46	9.97 ± 16.73	9.94 ± 7.20	14.5 ± 23.4	0.02^##^
Median (25^th^ percentile, 75^th^ percentile)	8.91 (6.13; 12.00)	8.59 (5.90; 11.35)	9.10 (6.42; 12.64)	10.84 (7.80; 15.16)	
Intima-media-thickness (mm)					
Mean ± SD	0.85 ± 0.14	0.82 ± 0.13	0.91 ± 0.14	0.92 ± 0.14	0.19^##^
Median (25^th^ percentile, 75^th^ percentile)	0.83 (0.74; 0.93)	0.80 (0.73; 0.89)	0.90 (0.80; 1.01)	0.90 (0.82; 1.02)	

All analyses are adjusted for age and sex.

*Abbreviations*: *NGT* normal glucose tolerance, *T2D* type 2 diabetes mellitus, *BMI* body mass index, *MI* myocardial infarction, *hsCRP* high sensitive C-reactive protein (serum).

^a^Mean ± standard deviation, median (25^th^ percentile, 75^th^ percentile), proportions (%).

^b #^F-Test; ^##^log F-Test; ^###^logistic regression.

^c^High alcohol intake: ≥ 40 g/day in men, ≥ 20 g/day in women.

^d^Systolic blood pressure ≥ 140/90 mm Hg or antihypertensive medication.

^e^Less than 1 hour of physical activity per week.

**Table 2:** **Spearman correlation coefficients (p values) between copeptin levels and cardiovascular risk factors (n = 1596)**

	**Total sample (n = 1596)**	**Men (n = 780)**	**Women (n = 816)**
**Age**	**0.187 (<0.001)**	**0.217 (<0.001)**	**0.157 (<0.001)**
**BMI**	**0.106 (<0.001)**	-0.020 (0.57)	**0.139 (<0.001)**
**Waist circumference**	**0.201 (<0.001)**	0.014 (0.69)	**0.126 (<0.001)**
**Systolic blood pressure**	**0.139 (<0.001)**	0.014 (0.70)	**0.080 (0.02)**
**Diastolic blood pressure**	**0.051 (0.04)**	-0.056 (0.12)	0.002 (0.94)
**Triglycerides**	**0.094 (<0.001)**	-0.020 (0.58)	**0.078 (0.03)**
**HDL cholesterol**	**-0.135 (<0.001)**	0.011 (0.75)	-0.054 (0.12)
**hsCRP**	**0.139 (<0.001)**	0.115 (0.001)	**0.179 (<0.001)**
**eGFR**	**-0.142 (<0.001)**	**-0.200 (<0.001)**	**-0.156 (<0.001)**

### Association of copeptin levels with IMT

Analyses of the correlations between IMT and copeptin were performed separately in men and women. Unadjusted copeptin levels were not associated with IMT in men (r = 0.04; p = 0.312) and only weakly associated with IMT in women (r = 0.14; p <0.001). Adjustment for age, for age and BMI, and multiple linear regression analyses using copeptin as independent variable resulted in a significant inverse relation between plasma copeptin levels and IMT in men (β = -0.035 mm, 95% CI: -0.061 mm; -0.009 mm; Q4 vs Q1 after multivariable adjustment). In the fully adjusted model, IMT was 0.038 mm (95% CI: -0.057; -0.020) lower in copeptin quartiles 3–4 than in quartiles 1–2. In the total cohort, copeptin and IMT were inversely associated after multivariable adjustment (β = -0.020 mm, 95% CI: -0.037 mm; -0.003 mm; Q4 vs Q1). IMT values of subjects with copeptin in quartiles 3–4 were 0.022 mm (95% CI: -0.034; -0.010) lower than in subjects in quartiles 1–2. Using copeptin as continuous variable resulted in a significant negative relation between copeptin levels and IMT in men after multivariable adjustment (Table 3). In contrast, in women, no significant association of copeptin and IMT was detectable in any of the applied regression models. Exclusion of study participants with former myocardial infarction or stroke or an eGFR < 60 ml/min from the analyses resulted in similar findings as in the entire study cohort (Additional file 2: Table S2).

**Table 3:** Association of plasma copeptin levels and IMT

	**Copeptin**	**Copeptin**	**Copeptin**	**β per 1 SD increase in copeptin**
	**Q3-4 vs Q1-2**	**Q4 vs Q1-Q3**	**Q4 vs Q1**	
**No adjustment**				
All subjects (N = 1,275)	0.010	**0.023**	**0.028**	0.005
	(-0.005; 0.026)	**(0.005; 0.041)**	**(0.006; 0.050)**	(-0.003; 0.013)
Men (N = 624)	-0.013	0.016	0.009	0.002
	(-0.036; 0.011)	(-0.011; 0.043)	(-0.024; 0.042)	(-0.010; 0.014)
Women (N = 651)	**0.032**	**0.030**	**0.046**	0.008
	**(0.012; 0.052)**	**(0.007; 0.053)**	**(0.018; 0.074)**	(-0.002; 0.018)
**Adjusted for age and sex**				
All subjects	**-0.018**	-0.011	-0.015	-0.001
	**(-0.030; -0.006)**	(-0.025; 0.003)	(-0.033; 0.002)	(-0.007; 0.005)
Men	**-0.042**	**-0.028**	**-0.038**	-0.009
	**(-0.060; -0.023)**	**(-0.049; -0.006)**	**(-0.064; -0.011)**	(-0.018; 0.001)
Women	0.005	0.004	0.006	0.005
	(-0.011; 0.020)	(-0.013; 0.022)	(-0.016; 0.027)	(-0.002; 0.013)
**Adjusted for age, sex and BMI**				
All subjects	**-0.018**	-0.012	-0.016	-0.002
	**(-0.030; -0.006)**	(-0.026; 0.002)	(-0.033; 0.0005)	(-0.008; 0.004)
Men	**-0.039**	**-0.026**	**-0.035**	-0.009
	**(-0.057; -0.020)**	**(-0.048; -0.005)**	**(-0.061; -0.009)**	(-0.018; 0.0002)
Women	0.002	0.002	0.003	0.004
	(-0.013; 0.018)	(-0.016; 0.020)	(-0.019; 0.024)	(-0.003; 0.012)
**Multivariable adjustment**^**a**^				
All subjects	**-0.022**	**-0.014**	**-0.020**	-0.003
	**(-0.034; -0.010)**	**(-0.028; -0.0002)**	**(-0.037; -0.003)**	(-0.009; 0.003)
Men	**-0.038**	**-0.024**	**-0.035**	**-0.010**
	**(-0.057; -0.020)**	**(-0.046; -0.002)**	**(-0.062; -0.009)**	**(-0.020; -0.001)**
Women	-0.002	-0.001	-0.001	0.004
	(-0.017; 0.014)	(-0.019; 0.017)	(-0.023; 0.021)	(-0.004; 0.012)

Gender-specific adjusted β’s (95% CI) for IMT given in mm as dependent variable and categories of copeptin as independent variable (highest quartile Q4 or Q3-4 versus lower quartiles (reference)): Results of linear regression models.

^a^Adjusted for: age, sex, BMI, waist (continuous), hypertension (systolic blood pressure ≥ 140/90 mm Hg or antihypertensive medication), HDL cholesterol (continuous), LDL cholesterol (continuous), triglycerides (continuous), former myocardial infarction or stroke, smoking (active/former/never), alcohol consumption (abstinent/moderate/high), physical activity (high/low), hsCRP, eGFR, prediabetes (yes/no), T2D (yes/no).

bold: p < 0.05.

### Relation of copeptin with IMT depending on glucose homeostasis

Median levels of copeptin were higher in subjects with T2D (10.8 (7.8; 15.2) pmol/l) compared to prediabetes (9.1 (6.4; 12.6) pmol/l) and NGT (8.6 (5.9; 11.4) pmol/l) (Table 1). Mean IMT was 0.082 ± 0.016 mm and 0.097 ± 0.016 mm higher in women and men with T2D as compared to individuals with NGT (p < 0.0001). This association disappeared after multivariable adjustment for conventional cardiovascular risk factors. A significant inverse correlation of IMT and copeptin was apparent in normoglycemic subjects and in study participants with prediabetes after adjustment for age, sex and BMI and after multivariable adjustment (Table 4). Using copeptin as continuous variable resulted in a significant negative relation between copeptin levels and IMT in study participants with prediabetes after adjustment for age and sex, for age, sex and BMI and after multivariable adjustment (Table 4). There was no significant association of copeptin and IMT in patients with overt T2D.

**Table 4:** Association of copeptin levels and IMT according to categories of glucose homeostasis

	**Copeptin**	**Copeptin**	**Copeptin**	**β per 1 SD increase in copeptin**
	**Q3-4 vs Q1-2**	**Q4 vs Q1-Q3**	**Q4 vs Q1**	
**Total study cohort**				
**No adjustment**				
NGT (n = 911)	0.007	0.019	0.024	0.001
	(-0.010; 0.025)	(-0.002; 0.040)	(-0.001; 0.049)	(-0.008; 0.010)
Prediabetes (n = 224)	-0.010	-0.009	-0.024	-0.011
	(-0.046; 0.027)	(-0.049; 0.031)	(-0.075; 0.027)	(-0.055; 0.032)
T2D (n = 140)	-0.018	-0.003	0.001	0.009
	(-0.068; 0.032)	(-0.051; 0.046)	(-0.076; 0.077)	(-0.006; 0.024)
**Adjustment for age and sex**				
NGT	**-0.015**	-0.004	-0.007	-0.004
	**(-0.029; -0.002)**	(-0.020; 0.012)	(-0.026; 0.012)	(-0.011; 0.002)
Prediabetes	-0.028	**-0.040**	**-0.058**	**-0.044**
	(-0.060; 0.004)	**(-0.075; -0.005)**	**(-0.103; -0.014)**	**(-0.082; -0.006)**
T2D	-0.025	-0.009	-0.008	0.010
	(-0.071; 0.020)	(-0.054; 0.035)	(-0.078; 0.062)	(-0.003; 0.024)
**Adjusted for age, sex and BMI**				
NGT	**-0.016**	-0.006	-0.008	-0.005
	**(-0.029; -0.003)**	(-0.022; 0.010)	(-0.027; 0.011)	(-0.012; 0.002)
Prediabetes	-0.027	**-0.038**	**-0.057**	**-0.042**
	(-0.058; 0.005)	**(-0.073; -0.004)**	**(-0.101; -0.013)**	**(-0.079; -0.004)**
T2D	-0.025	-0.008	-0.007	0.010
	(-0.070; 0.021)	(-0.053; 0.037)	(-0.078; 0.063)	(-0.003; 0.024)
**Multivariable adjustment**^**a**^				
NGT	**-0.018**	-0.007	-0.009	-0.006
	**(-0.031; -0.005)**	(-0.023; 0.008)	(-0.028; 0.010)	(-0.013; 0.0002)
Prediabetes	-0.031	**-0.041**	**-0.066**	**-0.050**
	(-0.064; 0.002)	**(-0.078; -0.005)**	**(-0.112; -0.019)**	**(-0.091; -0.009)**
T2D	-0.030	-0.009	-0.018	0.012
	(-0.081; 0.020)	(-0.059; 0.040)	(-0.096; 0.060)	(-0.002; 0.026)

Gender-specific adjusted β’s (95% CI) according to categories of glucose tolerance for IMT as dependent variable and categories of copeptin as independent variable (highest quartile Q4 or Q3-4 versus lower quartiles (reference)): Results of linear regression models.

^a^Adjusted for: age, sex, BMI, waist (continuous), hypertension (systolic blood pressure ≥ 140/90 mm Hg or antihypertensive medication), HDL cholesterol (continuous), LDL cholesterol (continuous), triglycerides (continuous), former myocardial infarction or stroke, smoking (active/former/never), alcohol consumption (abstinent/moderate/high), physical activity (high/low), hsCRP, eGFR.

bold: p < 0.05.

## Discussion

### Inverse association of copeptin levels with IMT

The key finding of the present study is a significant inverse relation of copeptin plasma levels with IMT in a community-based cohort. Our data suggest that moderately increased copeptin concentrations may indicate a reduced risk of early carotid atherosclerotic lesions in men. Our finding is unexpected, since AVP was considered to exert mainly proatherogenic effects and previous studies suggest a link between AVP/copeptin and the development of vascular complications [9-11,16-18]. Copeptin has negative prognostic value for all-cause mortality in severe disorders, such as heart failure [8,30-32], coronary artery disease [33,34], acute myocardial infarction [35] and stroke [17], and end-stage renal disease [14], whereas, to our knowledge, copeptin has not been described as a risk factor for future cardiovascular events in stable patients. Plasma copeptin levels do not reflect coronary artery status in stable patients with coronary artery disease [36,37] or predict cardiovascular events and incident heart failure in population-based studies [37-39]. In line with these results our study demonstrates that circulating copeptin is not an indicator for early atherosclerosis.

What is a possible explanation for the observed inverse association between copeptin and IMT? AVP is not only involved in vasoconstriction but also in nitric oxide-mediated vasorelaxation through the binding to the oxytocin receptor on vascular endothelium [2,40]. Experimental studies showed that AVP infusion in normotensive rats results not only in blood pressure increase, but also a V1R-mediated active vasodilatation of carotid arteries, probably mediated by endothelial release of nitric oxide [40], which plays an essential role in protection against vascular inflammation and endothelial dysfunction. The net effect of circulating AVP on the vascular bed may depend on the absolute hormone concentration [2]. In the present study, the median copeptin level was 8.6-9.1 pmol/l in non-diabetic individuals, which is comparable to levels described in other population-based studies (ranging from 5.0 pmol/l to 8.2 pmol/l [4,6,7,41]), but 2-10-fold lower than reported in studies investigating severe disorders, such as stroke, myocardial infarction or end stage kidney disease [9,14,42]. High AVP plasma concentrations predominantly stimulate V1aR, mediating vasoconstriction [43] and possibly increasing the prothrombotic potential by platelet activation [44]. Whereas high AVP levels are proatherogenic or indirectly indicate an increased risk for fatal complications, intermediate AVP concentrations may exert an inert or even protective effect on vascular endothelium. This hypothesis needs to be confirmed in future experimental studies.

So far, only few studies have investigated the association between copeptin concentrations and the development and progression of chronic atherosclerosis in the general community. One study found elevated copeptin concentrations to be associated with a decreased ankle-brachial index in patients with peripheral arterial disease. However, this association was only significant in African-Americans, but not in non-Hispanic whites, and all study participants had a family history of hypertension [45]. Another study described a positive association between copeptin levels and IMT in middle-aged individuals with chronic kidney disease [46] and a further study reported that copeptin is a predictor of stroke in hemodialysis patients [14]. Additionally, high copeptin levels predict microalbuminuria, an early sign of target organ damage in diabetes and hypertension [24,47]. However, high AVP plasma levels probably promote microalbuminuria primarily via V2R, a pathway not necessarily connected to atherosclerotic changes. Moreover, patients with renal insufficiency differ from the present population in several aspects. Chronic kidney disease patients are at high risk for cardiovascular events, often suffer from disturbances of sodium homeostasis and may have an overly activated AVP system. In addition, kidney function has a major impact on copeptin clearance [48]. Therefore, the discrepant results may be explained by different study population characteristics.

The effect of elevated AVP levels may depend on the receptor subtype distribution and regulation. AVP has been shown to exert certain pathophysiological effects in a gender-specific manner, e.g. a higher anti-diuretic response in females, probably due to a higher V2R density in the collecting duct and a decreased blood and pulse pressure sensitivity [49,50]. While, in rats, AVP induces a stronger contraction of coronary arteries in males [51], maximal contraction of the aorta in response to vasopressin is about threefold higher in females due to a weaker endothelium-derived nitric oxide-mediated vasodilatation [52]. In addition, potentially protective vasopressin effects seem to be more present in men. For example, a V2-like receptor related vasodilator effect exists in male rats, but not in females [53]. Thus, females seem to be less well protected against harmful vascular AVP. This might be one explanation for the lacking relationship between copeptin and IMT in women.

### Relation of copeptin with IMT depending on glucose homeostasis

After stepwise adjustment for age and sex, for age, sex and BMI and in the multivariate model, copeptin displayed significant associations with IMT only in subjects with NGT and with disturbed glucose tolerance, but not in patients with overt T2D. As described in previous studies [5-7], we found an increase of plasma copeptin in patients with T2D. Assuming that at least part of diabetic patients have high fasting blood glucose, the observed attenuation of the relation between copeptin and IMT may be explained to some extent by a slightly increased plasma osmolality followed by activation of the AVP system. It is tempting to speculate that the osmolality-mediated AVP/copeptin release might result in hormone concentrations that are above the threshold value predominantly mediating protective vascular effects. Alternatively, additional risk factors may be present in diabetic patients, overriding the potential protective effect of copeptin. It is important to note that we observed a trend towards an inverse correlation between plasma copeptin and IMT also in patients with T2D, suggesting that elevated copeptin is not a risk factor for early carotid atherosclerosis in patients with T2D. This interpretation is in agreement with a recent prospective study in diabetic patients showing that copeptin is related to all-cause mortality but only non-significantly associated with cardiovascular events [19].

### Study limitations

The major limitation of our study is its cross-sectional design, precluding statements on causalities. Furthermore, although multivariable adjustments were performed for all traditional cardiovascular risk factors and other potentially confounding parameters, residual confounding cannot be excluded.

## Conclusions

We provide the first data on the association of copeptin with IMT in a large population-based cohort stratified by gender, impaired glucose tolerance and T2D. We found an inverse association of copeptin and IMT, suggesting that moderately increased copeptin concentrations are related to a lower degree of atherosclerotic alterations in the CCA. Thus, plasma copeptin does not seem to be an independent risk factor for early carotid atherosclerosis in the general population. Whether slightly increased copeptin concentrations may be protective from macrovascular complications needs to be investigated in a prospective epidemiological study.

## Abbreviations

AVP: Arginine vasopressin; BMI: Body mass index; CCA: Common carotid artery; CI: Confidence interval; eGFR: Estimated glomerular filtration rate; HDL: High-density lipoprotein; hsCRP: High sensitive C-reactive protein; IMT: Intima-media thickness; KORA: Cooperative Health Research in the Region of Augsburg; LDL: Low-density lipoprotein; MI: Myocardial infarction; NGT: Normal glucose tolerance; OR: Odds ratio; Q: Quartile; r: Sperman correlation coefficient; T2D: Type 2 diabetes; VR: Vasopressin receptor; vs: Versus.

## Competing interests

The authors report that there is no duality of interest associated with this manuscript.

## Authors’ contribution

CT, BK, AL, CM, AP, WR and JS participated in the conception and design of the study; CT, CM, MH, WK, AP, JT, WR, JS collected the data; CT, BK, WR and JS analyzed the data; CT, BK, AL, CM, MH, WK, AP, JT, WR and JS participated in the interpretation of the results and the writing of the manuscript. All authors read and approved the final manuscript.

## Supplementary Material

Additional file 1: Table S1 Copeptin values in the respective quartiles. Click here for file

Additional file 2: Table S2 Relation of plasma copeptin and IMT. Gender-specific adjusted β’s (95% CI) for IMT given in mm as dependent variable and categories of copeptin as independent variable (highest quartile Q4 versus lowest quartile Q1): Results of linear regression models excluding study participants with former myocardial infarction or stroke or with an eGFR < 60 ml/min. Click here for file
