# Supplementary material for: Plasma copeptin levels are inversely associated with intima-media-thickness in men: the population-based KORA F4 study
Source: Cardiovasc Diabetol. 2013 Nov 11;12:168. doi: 10.1186/1475-2840-12-168 (PMC3827501; doi:10.1186/1475-2840-12-168)
Supplement: Additional file 1: Table S1 — Copeptin values in the respective quartiles. [file 1475-2840-12-168-S1.doc]

**Additional Table 1**

**Copeptin values in the respective quartiles.**

| **Total study cohort (n = 1275)** | **n** | **Mean ± SD** | **Median (q1; q3)** |
| --- | --- | --- | --- |
| quartile 1 | 317 | 2.22 ± 2.47 | 0.40 (0.40; 5.36) |
| quartile 2 | 319 | 7.45 ± 0.83 | 7.43 (6.73; 8.18) |
| quartile 3 | 320 | 10.29 ± 0.87 | 10.28 (9.50; 11.02) |
| quartile 4 | 319 | 21.83 ± 29.53 | 15.49 (13.43; 19.13) |
| **Men (n = 624)** | **n** | **Mean ± SD** | **Median (q1; q3)** |
| quartile 1 | 156 | 4.17 ± 2.89 | 5.61 (0.40; 6.53) |
| quartile 2 | 156 | 8.81 ± 0.78 | 8.96 (8.14; 9.44) |
| quartile 3 | 156 | 11.60 ± 1.10 | 11.44 (10.58; 12.65) |
| quartile 4 | 156 | 25.31 ± 32.76 | 17.36 (15.35; 21.04) |
| **Women (n = 651)** | **n** | **Mean ± SD** | **Median (q1; q3)** |
| quartile 1 | 162 | 0.78 ± 1.27 | 0.40 (0.40; 0.40) |
| quartile 2 | 163 | 6.35 ± 0.64 | 6.38 (5.78; 6.87) |
| quartile 3 | 161 | 8.99 ± 0.88 | 8.91 (8.24; 9.74) |
| quartile 4 | 165 | 17.85 ± 25.73 | 12.95 (11.38; 15.94) |
