# Supplementary material for: Plasma copeptin levels are inversely associated with intima-media-thickness in men: the population-based KORA F4 study
Source: Cardiovasc Diabetol. 2013 Nov 11;12:168. doi: 10.1186/1475-2840-12-168 (PMC3827501; doi:10.1186/1475-2840-12-168)
Supplement: Additional file 2: Table S2 — Relation of plasma copeptin and IMT. Gender-specific adjusted β’s (95% CI) for IMT given in mm as dependent variable and categories of copeptin as independent variable (highest quartile Q4 versus lowest quartile Q1): Results of linear regression models excluding study participants with former myocardial infarction or stroke or with an eGFR < 60 ml/min. [file 1475-2840-12-168-S2.doc]

**Additional Table 2**

**Relation of plasma copeptin and IMT**. Gender-specific adjusted β´s (95% CI) for IMT given in mm as dependent variable and categories of copeptin as independent variable (highest quartile Q4 versus lowest quartile Q1): Results of linear regression models excluding study participants with former myocardial infarction or stroke or with an eGFR < 60 ml/min.

|  | **Copeptin**  **Q4 vs Q1** |
| --- | --- |
| **No adjustment** |  |
| All subjects (N=1,143) | 0.019  (-0.004; 0.042) |
| Men (N=551) | 0.001  (-0.034; 0.037) |
| Women (N=592) | **0.036**  **(0.006; 0.065)** |
| **Adjusted for age and sex** | |
| All subjects | -0.015  (-0.033; 0.002) |
| Men | **-0.033**  **(-0.061; -0.005)** |
| Women | 0.001  (-0.021; 0.024) |
| **Adjusted for age, sex and BMI** | |
| All subjects | -0.016  (-0.033; 0.0002) |
| Men | **-0.030**  **(-0.057; -0.002)** |
| Women | -0.001  (-0.023; 0.021) |
| **Multivariable adjustment^a^** | |
| All subjects | **-0.019**  **(-0.037; -0.002)** |
| Men | **-0.031**  **(-0.058; -0.003)** |
| Women | -0.004  (-0.026; 0.019) |

^a^ adjusted for: age, sex, BMI, waist (continuous), hypertension (systolic blood pressure ≥ 140/90 mm Hg or antihypertensive medication), HDL cholesterol (continuous), LDL cholesterol (continuous), triglycerides (continuous), smoking (active/former/never), alcohol consumption (abstinent/moderate/high), physical activity (high/low), hsCRP, eGFR, prediabetes (yes/no), T2D (yes/no).
